# Pleth variability index and fluid management practices: a multicenter service evaluation

**DOI:** 10.1186/s13104-021-05705-6

**Published:** 2021-07-28

**Authors:** Patrice Forget, Simon Lacroix, Eric P. Deflandre, Anne Pirson, Nicolas Hustinx, Olivier Simonet, Fabrice Wandji, Serge von Montigny, Jibba Amraoui

**Affiliations:** 1 Institute of Applied Health Sciences, Epidemiology group, School of Medicine, Medical Sciences and Nutrition, University of Aberdeen, NHS Grampian, Department of Anaesthesia, Aberdeen, UK; 2 Clinique Saint-Luc of Bouge, Department of Anaesthesia, Extracorporeal Circulation Team, Namur, Belgium; 3 Clinique Saint-Luc of Bouge, Department of Anaesthesia, Namur, Belgium; 4 Cabinet Medical ASTES, Jambes, Belgium; 5 University of Liege, Liege, Belgium; 6 Grand Hopital de Charleroi, Charleroi, Belgium; 7 Centre Hospitalier de Wallonie Picarde, Department of Anaesthesia, Tournai, Belgium; 8 Centre Hospitalier Mons-Warquignies, Department of Anaesthesia, Mons, Belgium; 9 Institut du Cancer, Montpellier, France

## Abstract

**Objectives:**

The introduction of a new technology has the potential to modify clinical practices, especially if easy to use, reliable and non-invasive. This observational before/after multicenter service evaluation compares fluid management practices during surgery (with fluids volumes as primary outcome), and clinical outcomes (secondary outcomes) before and after the introduction of the Pleth Variability Index (PVI), a non-invasive fluid responsiveness monitoring.

**Results:**

In five centers, 23 anesthesiologists participated during a 2-years period. Eighty-eight procedures were included. Median fluid volumes infused during surgery were similar before and after PVI introduction (respectively, 1000 ml [interquartile range 25–75 [750–1700] and 1000 ml [750–2000]). The follow-up was complete for 60 from these and outcomes were similar. No detectable change in the fluid management was observed after the introduction of a new technology in low to moderate risk surgery. These results suggest that the introduction of a new technology should be associated with an implementation strategy if it is intended to be associated with changes in clinical practice.

**Supplementary Information:**

The online version contains supplementary material available at 10.1186/s13104-021-05705-6.

## Introduction

**Fig. 1:**
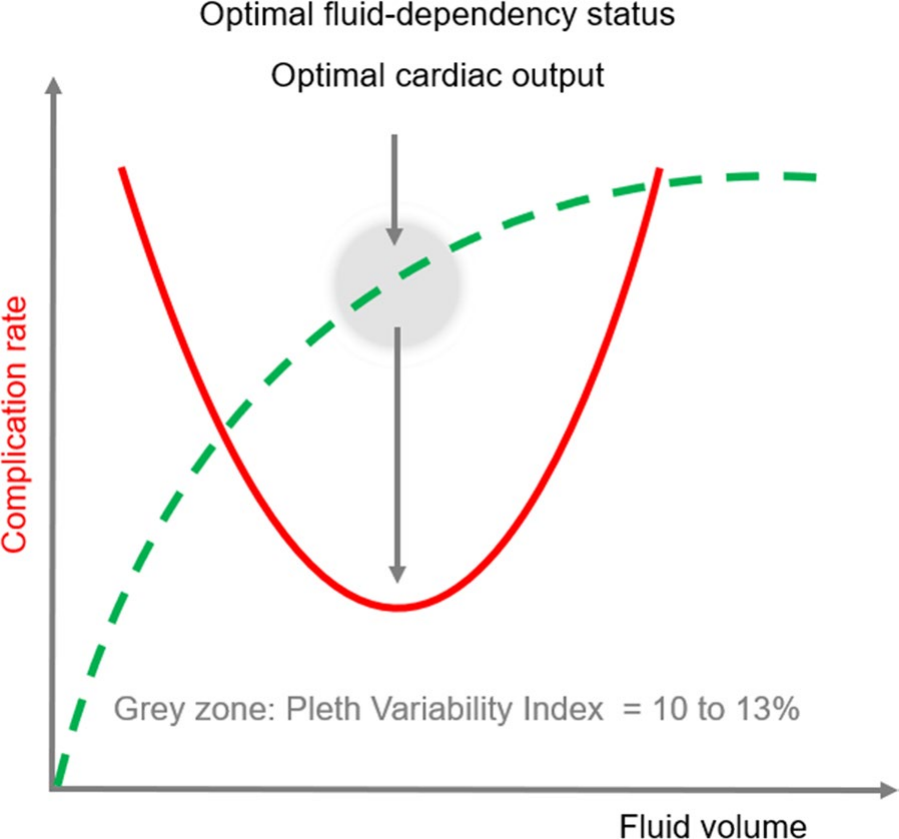
Relationship between complication rate, fluid volume administered during surgery. Hypo- (on the left of the U-curve) as well as hypervolemia (on the right) may be associated with postoperative complications. Fluid management associated with optimal cardiac output (green curve) may correlate with the lower rate of perioperative complications

The management of intraoperative blood volume and the major complications of both hypovolemia and hypervolemia remain major and unresolved issues [1]. It has been suggested that dynamic parameters such as pulse pressure or stroke volume variation may, at least partially, solve this problem [2]. Dynamic parameters assess hemodynamic response to respiratory variations which allows to monitor the fluid-dependency status. They have been well validated for the prediction of the response to a fluid challenge [2]. The Pleth Variability Index (PVI), as a dynamic and non-invasive parameter, could fill a gap for the low to moderate risk patient and procedure, especially when cardiac output monitoring is not considered [2]. To improve fluid management, the optimization of the PVI value has been proposed [3, 4] by targeting a PVI value between 10 and 13%. Optimizing this value may help optimize cardiac output without taking the risk of giving too much fluids (i.e. fluids not associated with a significant increase in cardiac output) (Fig. 1). However, the current ability of the introduction of PVI on its own to change the practice of clinicians is not known.

This multicenter service evaluation aims to establish whether the introduction of the PVI in the clinical practice is associated with a modification of the practice during low to moderate risk surgery.

## Main text

### Subjects and methods

#### Design and outcomes

This before/after multicenter service evaluation compares the fluid volumes used before and after the introduction of the PVI, focusing on fluids volumes (primary outcome), with clinical outcomes as quality indicators (secondary outcomes).

Thus, three consecutive phases were planned: a pilot survey (preparatory phase), a phase 1 (before the introduction of the PVI) and a phase 2 (after).

This work is presented following the Strengthening the Reporting of Observational Studies in Epidemiology (STROBE) Statement for cohort studies [5].

#### Ethics committee consideration

This multicenter service evaluation aims to assess how clinicians change their fluid management after the introduction of the PVI in three kind of surgery: Knee/hip arthroplasty, colorectal surgery. Written informed consent was waived by the ethics committee of the principal investigator (Patrice Forget, affiliated to the UCLouvain in 2012) (26th March 2012, Chairperson: Prof. J-M Maloteaux, Commission d'Ethique Biomédicale Hospitalo- Facultaire, Institution: UCLouvain), because considered as a practices audit, the essential information to be recorded being physicians’ practices changes, without any breach in patient confidentiality. The project was registered before any data collection (Clinicaltrials.gov: NCT02271841) and performed in accordance with the ethical standards of the Declaration of Helsinki (1964) and its subsequent amendments.

#### Settings

The recruitment of a minimum of 3 centers was anticipated in Belgium and/or France who had no significant experience with the technology, to reach approximately 30 participating anesthesiologists. All the centers (n = 10) that worked within the network of the university hospital were considered.

#### Inclusion / exclusion criteria

Fives sites were recruited to participate in this research; each site may be in use of Masimo technology, but not PVI at the time. More than one anesthesiologist per center was recommended. Into these centers, all anesthesiologists had the opportunity to participate.

The project was limited to surgical procedures like knee/hip arthroplasties and colorectal surgery. To avoid a too high heterogeneity challenging data interpretation, patients under 18 years and ASA (American Society of Anesthesiologists) 4 were excluded.

#### Equipment

Standard of care monitoring for surgery included a pulsoxymeter, continuous electrocardiogram, non-invasive blood pressure monitoring, gas analyzers including capnography. No limitation was suggested in the use of other type of monitoring.

The use of Masimo Radical 7 devices SET technology with PVI feature was possible after the introduction of the technology, which was preceded by an appropriate training (delivered by PF, proposing a grey zone approach for the decision of fluid loading above a PVI value of more than 10 to 13% during more than 5 min) [3, 4]. During this training, the determinants of cardiac output and the influence of intravascular blood volume were discussed. The advantages and disadvantages of the different types of advanced hemodynamic monitoring were presented and discussed with the participants. The information was based on a previously published expert consensus [2].

#### Statistical analysis

Based on previously published data in similar procedures, with low expected variability, a difference of 250 ml ± 250 ml could be anticipated [4]. This small difference was chosen to unmask any practice change, small or large in the fluid management, independently on any clinical significance. The type of surgery was considered for low variability, rather than being able to measure improvement in outcome. The clinical results were considered as secondary outcomes, forming part of a service evaluation. For this, we used the Dindo-Clavien and the Postoperative Morbidity Survey classifications [6, 7].

A sample size calculation showed that at least 22 procedures recorded before and after (44 in total) would be sufficient to exclude the null hypothesis (being the absence of a difference of 250 ml ± 250 ml) with an alpha of 0.05 and a power of 90% [4]. We decided not to restrict the number of procedures, up to end 2018.

Data were collected and managed using REDCap (REsearch Electronic Data Capture) tools [8]. The forms were designed by the research team and tested with dummy data before use. Non-response bias was minimized as much as possible through automatic reminders and personal communication from the lead investigator. There was no incentive other than to receive the material for free and be part of the research project.

As normal distribution was rejected in most cases, Mann–Whitney U test was used for the comparisons. Data are given with mean ± SD or median [interquartile range 25–75] [IQR25-75], and 95% confidence interval (95%CI) as appropriate. A p-value of less than 0.05 was considered significant. No subgroup analysis, sensitivity nor other inferential analysis was planned. Statistica version 7.0 (STATSOFT, Tusla, USA) was used for all the analyses.

### Results

#### Pilot survey

A pilot survey was sent to 19 anesthesiologists to document the current practices (Additional file 1: Tables S1, S2, Figure S1). These nineteen were all from the 23 ultimately included who accepted to respond to this pilot questionnaire. This confirmed that an advanced and/or invasive monitoring was frequently used by most of the practitioners.

#### Participants

After the pilot survey, 75 anesthesiologists were approached, in five centers, by the local investigator and 23 completed the project (Additional file 1: Fig. S1). The reasons for not including the others were not documented. These practitioners progressively started the first phase (before the introduction of the new monitoring), including consecutive patients from February 2015 up to October 2017. After the introduction of the PVI, the second phase of the data collection occurred for all the centers during the year 2018.

#### Patients and procedures

In the five centers, 88 patients and procedures were followed (Table 1). The follow-up was complete for 60 patients from the 88. Ventilation practices were slightly different (more frequently administered lower tidal volumes) but this was not statistically significant (Table 1).

**Table 1:** Characteristics of the 88 patients and procedures

	Valid N	Mean	Std.Dev	Median	Lower quartile	Upper quartile
Age (years)	88	68.5	9.5	68	63	75
Before	54	69.2	9.1	68	64	75
After	34	67.3	10.1	68	62	73
Sex: males; females	35;53					
Before	21;33					
After	13;21					
Height (cm)	86	165.4	9.9	165	158	172
Before	52	164.9	10.2	165	157	172
After	34	166.1	9.7	165	160	175
Weight (kg)	87	77.4	18.7	76	60	94
Before	53	77.8	20.8	77	60	95
After	34	76.8	15.2	76	67	84
ASA: 1; 2; 3; 4 (n)	7;56;16;0(/79					
Before	5;33;7;0(/45)					
After	2;23;9;0(/34)					
Length of the surgery (min)	85	136	76	120	90	180
Before	52	127	66	103	90	150
After	33	149	90	125	90	195
General anesthesia	62(/88)					
Before	43(/54)					
After	24(/34)					
TKP; THP; Colonic surgery	27;31;30(/88)					
Before	17;22;15(/54)					
After	10;9;15(/34)					
Laparoscopic approach (n)	29					
Before	14					
After	15					
Tidal volume (ml)	62	524	274	460	450	500
Before	39	559	338	500	450	525
After	23	464	70	450	400	475
Frequency at beginning (cpm)	63	14	2	14	12	14
Before	40	13	2	13	12	14
After	23	15	3	14	12	16

*ASA* American Society of Anesthesiology score, *TKP* Total knee prosthesis placement, *THP* Total hip prosthesis placement, Frequency at the beginning: refers to respiratory frequency at the beginning of the surgery, *cpm* cycles per minute

The total volume administered during surgery was similar when comparing the period before PVI introduction with the period after (primary outcome) (median [IQR25-75]: respectively, 1000 ml [750–1700] and 1000 ml [750–2000]). The other outcomes were also similar (Table 2, Additional file 1: Figure S2) (p > 0.05 for all the comparisons).

**Table 2:** Outcomes of the 88 patients and procedures

	Valid N	Median	Lower quartile	Upper quartile
Primary outcome				
IV volume administered (ml)	88	1000	750	1750
Before	54	1000	750	1700
After	34	1000	750	2000
Secondary outcomes				
Length of stay, ICU (days)	60	0.0	0.0	0.0
Before	35	0.0	0.0	0.0
After	25	0.0	0.0	0.0
Length of hospital stay (days)	60	5.0	4.0	6.0
Before	35	5.0	4.0	7.0
After	25	5.0	4.0	6.0
Postop. complications (Dindo-Clavien)				
(Grade 0;1;2;3;4;5)	60	38;17;3;2;0;0		
Before	35	21;9;3;2;0;0		
After	25	17;8;0;0;0;0		

IV: intravenous; ICU: Intensive care unit; Dindo-Clavien classification of postoperative complications: Grade 0: no complication. Grade I: Any deviation from the normal postoperative course without the need for pharmacological treatment or surgical, endoscopic and radiological interventions. Allowed therapeutic regimens are: drugs as antiemetics, antipyretics, analgesics, diuretics and electrolytes and physiotherapy. This grade also includes wound infections opened at the bedside. Grade II: Requiring pharmacological treatment with drugs other than such allowed for grade I complications. Blood transfusions and total parenteral nutrition are also included. Grade III: Requiring surgical, endoscopic or radiological intervention. Grade IV: Life-threatening complication. Grade V: Death of a patient

### Discussion

No detectable change occurred before and after the introduction of the PVI. Particularly, no change was observed in the average amounts of fluids used during prosthesis and colonic surgeries after this introduction of a fluid responsiveness monitoring. However, this does not permit to exclude any change in the practice that may occur in particular patients, but not visible in aggregated analyses.

Moreover, the changes in practices may be limited by several factors, notably a probable evolution in the ventilatory practices, toward lower tidal volumes and slightly higher frequency, even if not statistically significant. Interestingly, the pilot survey showed that most of the practitioners declared that the institution does not have any written protocol, care guide or statement concerning hemodynamic management. This may introduce the question whether a new monitoring may help if not introduced in a protocol. An observation of this is the fact that, in a meta-analysis on goal-directed therapy (GDT), the effect of the intervention was particularly evident in older series, and namely in non-ERAS (enhanced recovery after surgery) programs [8]. One may speculate that the existence of a protocol (in this case, ERAS program) may have an important impact interacting with the added value of a new monitoring. These patients may also present a lower risk profile, and then benefiting less of a GDT. On the other hand, the introduction of a quality improvement program has been recently shown as complex, and not necessarily directly associated with improved outcomes [9]. The relative low introduction rate of the cardiac-output monitoring in the EPOCH trial reinforced our observation that invasive technologies would not be seen suitable in most low to moderate risk procedures [1].

The question remains whether a non-invasive technology may replace more invasive ones for low to moderate risk surgery. This study was not designed for, but the potential exists and would merit to be further explored. Exploratory analyses showed that 38/54 procedures (70%) before the introduction of the PVI did not reported any use of an invasive monitoring vs. 27/34 (79%) (p > 0.05) suggesting a shift toward less invasive monitoring.

In conclusion, this work shows that no detectable change in the fluid management was observed after the introduction of a new technology in low to moderate risk surgery.

## Limitations

The limitations of this work are linked to the small sample size, small tidal volumes, laparoscopic procedures, low risk surgery, and the lack of generalizability in other contexts, like in higher risk surgery, but highlight the fact that even in low variability procedures, there is a need for implementation of protocols and a place for non-invasive monitoring.

## Supplementary Information

**Additional file 1: Table S1.** Background of 19 anesthesiologists responding to a pilot survey regarding fluid management. IQR: interquartile range. **Table S2.** Practices of 19 anesthesiologists responding to a pilot survey regarding goal directed therapy (GDT) for fluid management. **Figure S1.** Flow chart. **Figure S2.** Total volume administered in 88 patients before and after the introduction of the Pleth Variability Index (expressed as median, 25–75 interquartile range and range). IV: intravenous.

## Data Availability

The data are available upon reasonable request addressed to the corresponding author (Patrice Forget, forgetpatrice@yahoo.fr).

## Abbreviations

PVI: Pleth Variability Index
STROBE: Strengthening the Reporting of Observational Studies in Epidemiology
ASA: American Society of Anesthesiologists
REDCap: REsearch Electronic Data Capture
SD: Standard deviation
IQR: Interquartile range
CI: Confidence interval
GDT: Goal-directed therapy
ERAS: Enhanced recovery after surgery
EPOCH trial: Enhanced Peri-Operative Care for High-risk patients trial
