# Supplementary material for: Pleth variability index and fluid management practices: a multicenter service evaluation
Source: BMC Res Notes. 2021 Jul 28;14:293. doi: 10.1186/s13104-021-05705-6 (PMC8317350; doi:10.1186/s13104-021-05705-6)
Supplement: Supplementary file 1 — Additional file 1: Table S1. Background of 19 anesthesiologists responding to a pilot survey regarding fluid management. IQR: interquartile range. Table S2. Practices of 19 anesthesiologists responding to a pilot survey regarding goal directed therapy (GDT) for fluid management. Figure S1. Flow chart. Figure S2. Total volume administered in 88 patients before and after the introduction of the Pleth Variability Index (expressed as median, 25–75 interquartile range and range). IV: intravenous. [file 13104_2021_5705_MOESM1_ESM.docx]

**APPENDIX**

**Tables**

| Age (median [IQR 25-75]) | 42 [38-52] |
| --- | --- |
| How long have you been providing or directly providing or directly 0-5 years supervising anesthesia? (0-5 years/6-10 years/More than 11 years) | (2/9/8) |
| Which best describes your current practice  (University Hospital / General Hospital / Private practice) | (3/12/4) |
| What is your familiarity of Goal Directed Therapy (GDT)? | (3/12/4) |
| Does your institution or group have a written protocol, care guide, or statement concerning hemodynamic management in this setting? (Yes / No) | (2/17) |

Table S1. Background of 19 anesthesiologists responding to a pilot survey regarding fluid management. IQR: interquartile range.

| **What monitoring do you routinely use for GDT for patients undergoing moderate to low risk surgery (frequency of use)?** | **<5 %** | **6-25%** | **26-50%** | **51-75%** | **>75%** |
| --- | --- | --- | --- | --- | --- |
| Global end diastolic volume | 18 | 1 | 0 | 0 | 0 |
| Central venous pressure | 16 | 1 | 1 | 1 | 0 |
| Invasive arterial pressure | 3 | 11 | 1 | 2 | 2 |
| Stroke Volume Variation | 11 | 3 | 3 | 1 | 1 |
| Mixed venous saturation (ScvO2) | 17 | 2 | 0 | 0 | 0 |
| Central venous saturation (SvO2) | 17 | 1 | 1 | 0 | 0 |
| Oxygen delivery (DO2) | 19 | 0 | 0 | 0 | 0 |
| Pulse Pressure Variation (invasive) | 9 | 7 | 3 | 0 | 0 |
| Pulmonary capillary wedge pressure | 18 | 0 | 0 | 1 | 0 |
| Transesophageal echocardiography | 12 | 4 | 1 | 1 | 1 |
| Non-invasive arterial pressure | 2 | 2 | 1 | 3 | 11 |
| Continuous non-invasive arterial pressure | 12 | 1 | 1 | 1 | 1 |
| Systolic Pressure Variation (invasive) | 7 | 6 | 5 | 0 | 1 |
| Cardiac output | 15 | 1 | 1 | 2 | 0 |
| Pleth Waveform Variation | 13 | 3 | 1 | 2 | 0 |

Table S2. Practices of 19 anesthesiologists responding to a pilot survey regarding goal directed therapy (GDT) for fluid management.

**Figures**


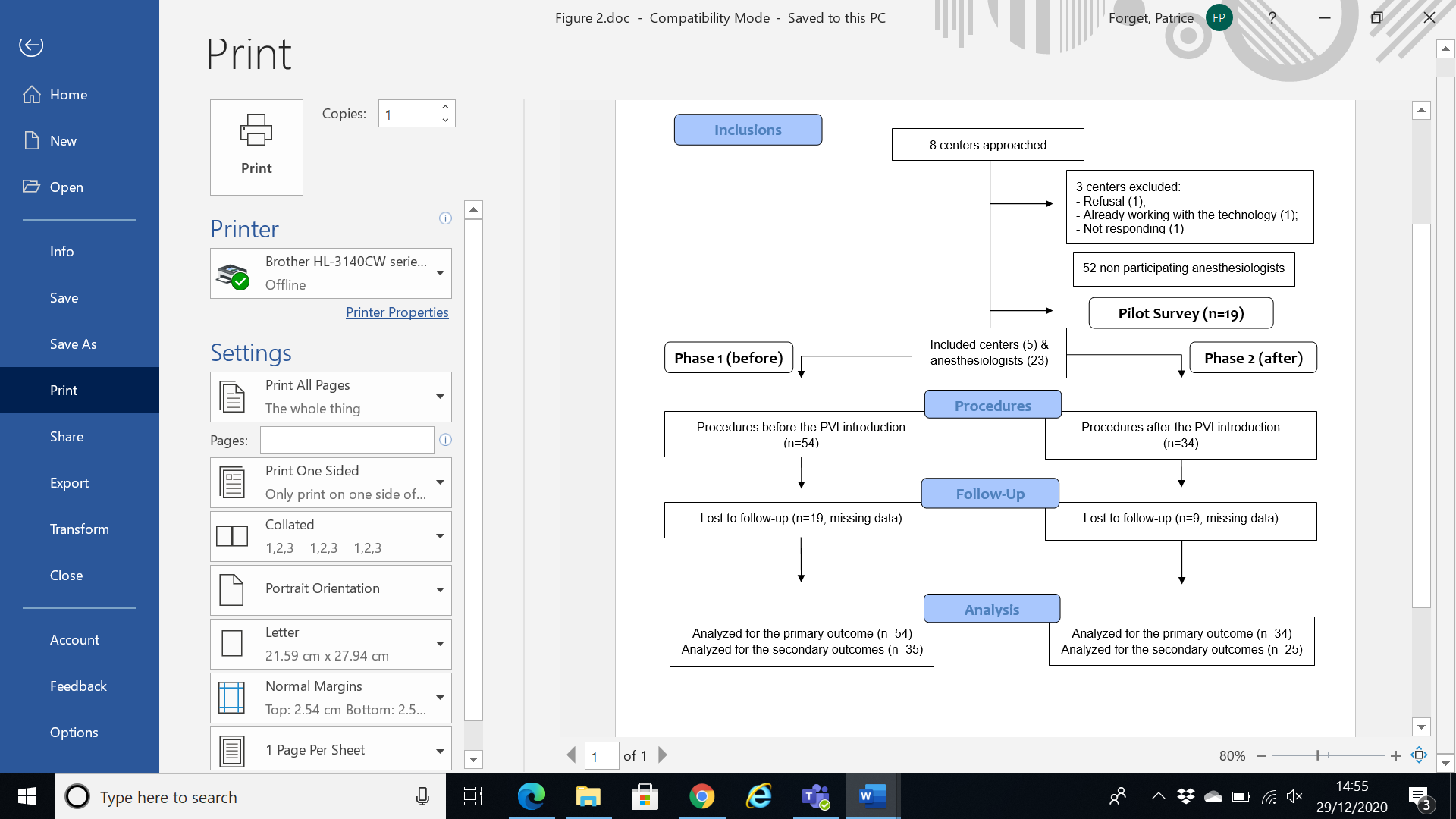


Figure S1. Flow chart.


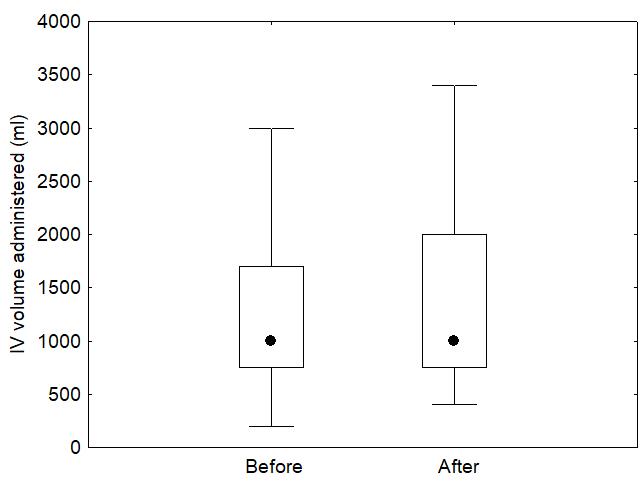


Figure S2. Total volume administered in 88 patients before and after the introduction of the Pleth Variability Index (expressed as median, 25-75 interquartile range and range). IV: intravenous.
